# Prevalence and Trends in Cigarette Smoking Among Adults with Epilepsy — United States, 2010–2017

**DOI:** 10.15585/mmwr.mm6947a5

**Published:** 2020-11-27

**Authors:** Sanjeeb Sapkota, Rosemarie Kobau, Janet B. Croft, Brian A. King, Craig Thomas, Matthew M. Zack

**Affiliations:** ^1^ASRT Inc., Atlanta, Georgia; ^2^Division of Population Health, National Center for Chronic Disease Prevention and Health Promotion, CDC; ^3^Office on Smoking and Health, National Center for Chronic Disease Prevention and Health Promotion, CDC.

Cigarette smoking remains the leading cause of preventable disease and death in the United States (*1*). Although the percentage of all U.S. adults who smoke cigarettes has declined substantially since the mid-1960s (*1*,*2*), marked disparities persist, and declines have not been consistent across population groups (*1*,*2*). Studies have shown that cigarette smoking is as common, and sometimes more so, among adults with a history of epilepsy compared with those without a history of epilepsy, but reasons for this are unclear (*3*–*6*). Compared with adults without epilepsy, adults with epilepsy report lower household income, more unemployment and disability, worse psychological health, and reduced health-related quality of life (*3*,*4*,*6*,*7*). Trends in cigarette smoking among U.S. adults with epilepsy have not been previously assessed. CDC analyzed National Health Interview Survey (NHIS) data among 121,497 U.S. adults from 2010, 2013, 2015, and 2017 to assess current cigarette smoking by epilepsy status. From 2010 through 2017, the age-standardized percentages of current smoking were 24.9% among adults with active epilepsy, 25.9% among adults with inactive epilepsy, and 16.6% among adults with no history of epilepsy. After accounting for differences in data collection intervals and patterns in smoking status among subgroups, CDC found that current cigarette smoking declined significantly from 2010 to 2017 among adults with no history of epilepsy (19.3% to 14.0% [p<0.001]) and inactive epilepsy (29.2% to 16.2% [p = 0.03]), but declines among adults with active epilepsy were not statistically significant (26.4% to 21.8% [p = 0.2]). Epilepsy health and social service providers should promote smoking cessation resources to adults with active epilepsy who smoke cigarettes to help them quit smoking and to reduce their risk of smoking-related disease and death.*

NHIS is an annual, nationally representative, in-person survey of the noninstitutionalized U.S. civilian population. The NHIS Sample Adult core questionnaire is administered to a randomly selected adult aged ≥18 years in each family within the selected household. Sample sizes and final response rates for sample adults in each of the 4 years were as follows: 2010 (27,157; 72.1%), 2013 (34,557; 61.2%), 2015 (33,672; 55.2%) and 2017 (26,742; 53.0%).^†^ Supplementary questions on epilepsy were added to the Sample Adult Core component of NHIS in 2010, 2013, 2015, and 2017.

Respondents were defined as having “any epilepsy” (either active or inactive epilepsy) or no history of epilepsy based on three questions.^§^ Those who reported doctor-diagnosed epilepsy and also reported taking antiseizure medication, having one or more seizures in the past year, or both were classified as having “active” epilepsy. Respondents were classified as having “inactive” epilepsy if they reported a history of epilepsy but were not taking medication for epilepsy and had not had a seizure in the past year. Current combustible cigarette smoking was defined as self-reported use of at least 100 cigarettes during the respondent’s lifetime and smoking “every day” or “some days” at the time of interview. Current cigarette smoking,^¶^ by epilepsy status, was assessed overall and by survey year; data from 2010, 2013, 2015, and 2017 were aggregated to provide more stable estimates of current cigarette smoking by sex, age, race/ethnicity, education, family income,** health insurance coverage at the time of survey, employment status, disability status, U.S. Census region, and presence or absence of serious psychological distress.

SAS (version 9.4; SAS Institute) and SUDAAN (version 11.0; RTI International), which accounted for the respondent sampling weights and the NHIS complex sample design, were used for the analysis. The aggregated analytical sample for this report included 121,497 adults with complete data on epilepsy and current cigarette smoking status. All reported differences among three or more groups were assessed using a Wald F test; differences between two subgroups were assessed using two-tailed t-tests. The threshold for statistical significance for all tests was p<0.05. Orthogonal polynomials, a statistical analysis to examine trends, was used to estimate the decline in percentage of adults who smoked from 2010 to 2017, accounting for unequal data collection intervals and different patterns in smoking prevalence among the three subgroups.

Among all U.S. adults, 1.1% had active epilepsy and 0.7% had inactive epilepsy. Current cigarette smoking prevalence was 24.9% for adults with active epilepsy, 25.9% for adults with inactive epilepsy, and 16.6% for adults without epilepsy (Table). Current cigarette smoking prevalence was higher among adults with active epilepsy than among those with no history of epilepsy overall and for both men and women; adults aged 35–54 or 55–64 years; non-Hispanic Whites and Other, non-Hispanic adults; adults with <12 or >12 years of education; adults with family incomes <100% or >300% of the federal poverty level; adults with health insurance; unemployed adults; and adults residing in the Northeast, the Midwest, or the South (Table). Among those without serious psychological distress, current cigarette smoking among adults with active epilepsy was higher (22.4%) than it was among adults without epilepsy (15.7%).

**TABLE:** Age-standardized* estimates of current smoking^†^ prevalence among adults, by epilepsy status and selected characteristics — United States, 2010, 2013, 2015 and 2017

Characteristic	Active epilepsy		Inactive epilepsy		No history of epilepsy	
	No.^§^	% (95% CI)	No.^§^	% (95% CI)	No.^§^	% (95% CI)
**Total (crude)**	**1,372**	**25.2 (22.4–28.2)**	**868**	**26.5 (23.1–30.3)**	**119,257**	**16.3 (15.9–16.7)**
**Total (age-standardized)**	**1,372**	**24.9 (22.1–28.0)**	**868**	**25.9 (22.6–29.6)**	**119,257**	**16.6 (16.2–16.9)**
**Sex**						
Men	607	24.1 (20.0–28.7)	345	27.5 (22.3–33.4)	53,346	18.5 (18.0–19.0)
Women	765	25.5 (21.9–29.5)	523	25.3 (21.2–29.8)	65,911	14.7 (14.2–15.1)
**Age group (yrs)**						
18–34	287	22.4 (16.9–29.1)	241	24.8 (18.7–32.1)	31,891	17.9 (17.3–18.5)
35–54	503	33.1 (28.2–38.5)	315	31.7 (25.938.1)	39,089	18.8 (18.2–19.4)
55–64	296	28.4 (22.1–35.7)	166	25.1 (18.6–23.9)	20,006	17.1 (16.4–17.8)
≥65	286	7.8 (5.1–11.7)	146	15.0 (9.7–22.6)	28,271	8.7 (8.2–9.1)
**Race/Ethnicity**						
White, non-Hispanic	919	27.2 (23.8–30.9)	610	27.1 (23.1–31.4)	73,561	18.8 (18.3–19.3)
Black, non-Hispanic	218	16.6 (10.9–24.5)	122	26.6 (18.0–37.3)	16,397	17.2 (16.4–18.0)
Hispanic	145	14.9 (9.6–22.4)	89	14.1 (8.4–22.8)	19,611	10.8 (10.2–11.4)
Other, non-Hispanic	90	32.6 (21.7–45.7)	47	35.7 (20.6–54.2)	9,688	11.8 (10.9–12.8)
**Education level (yrs)**						
<12	311	34.3 (27.5–41.9)	141	39.7 (30.8–49.3)	17,123	25.3 (24.2–26.4)
12	415	25.3 (20.4–30.9)	231	28.3 (22.1–35.6)	30,044	23.9 (23.1–24.6)
>12	629	20.8 (17.2–24.9)	488	21.3 (17.4–25.9)	71,614	12.0 (11.7–12.4)
**Family income**						
<100% of FPL	362	35.2 (28.8–42.2)	173	40.7 (32.3–49.7)	15,019	25.9 (24.8–26.9)
100%–200% of FPL	305	26.4 (20.3–33.6)	151	30.9 (21.8–41.9)	18,570	21.3 (20.4–22.2)
201%–300% of FPL	145	28.7(18.9–41.0)	101	25.2 (16.6–36.3)	15,413	18.1 (17.2–18.9)
>300% of FPL	559	18.9 (15.0–23.6)	443	20.9 (16.9–25.6)	70,254	14.2 (13.8–14.6)
**Insurance status**						
Uninsured	121	35.2 (24.5–46.4)	126	40.1 (29.9–51.3)	16,635	26.1 (24.8–27.4)
Insured	1,246	24.1 (21.1–27.3)	739	23.3 (19.9–27.0)	102,206	14.7 (14.3–15.1)
**Current employment**						
Employed	375	17.6 (13.4–22.7)	410	21.7 (16.8–27.5)	70,478	15.1 (14.7–15.5)
Retired	229	^—¶^	122	67.8 (63.3–71.9)	24,358	24.7 (15.6–36.8)
Disabled	612	29.0 (24.3–34.2)	216	32.9 (24.9–42.1)	8,411	32.8 (30.9–34.6)
Unemployed	66	41.8 (29.2–55.7)	55	35.6 (21.5–52.8)	5,565	27.2 (25.6–28.9)
Other (e.g., student or homemaker)	88	21.1 (12.3–33.9)	65	23.7 (15.0–35.3)	10,394	12.8 (11.9–13.8)
**U.S. Census region**						
Northeast	210	25.4 (19.3–32.6)	111	25.2 (18.2–33.8)	19,510	14.8 (14.1–15.6)
Midwest	301	30.9 (24.5–38.2)	219	27.0 (20.6–34.4)	25,860	19.6 (18.8–20.5)
South	542	25.1(20.9–29.7)	323	26.2 (21.0–32.1)	43,189	17.8 (17.1–18.5)
West	319	18.1 (13.1–24.5)	215	22.4 (16.1–30.3)	30,698	13.1 (12.5–13.7)
Serious psychological distress**						
No	1,091	22.4 (19.4–25.6)	775	23.9 (20.5–27.7)	111,432	15.7 (15.4–16.1)
Yes	213	43.7 (34.9–52.9)	76	44.5 (30.1–59.9)	4,293	38.2 (36.3–40.1)

**Abbreviations:** CI = confidence interval; FPL = federal poverty level.

*Age-standardized to the 2000 U.S. projected population, aged ≥18 years, using four age groups (18–34, 35–54, 55–64, and ≥65 years). All percentages are age-standardized except those for age groups and overall (crude).

^†^ Current smoking was defined by self-report of smoking at least 100 cigarettes during one’s lifetime and smoking every day or some days at the time of the interview.

^§^ Categories in subgroups might not sum to total because of missing responses for some variables.

^¶^ Suppressed because relative standard error was ≥0.30.

** Serious psychological distress was defined as a score ≥13 for responses to six questions based on the Kessler psychological distress scale about feelings of hopelessness, sadness, nervousness, restlessness, worthlessness, and feeling like everything is an effort in the past 30 days. Participants were asked to respond on a Likert scale ranging from “None of the time” (score = 0) to “All of the time” (score = 4). Responses were summed over the six questions; respondents with a score of ≥13 were coded as having serious psychological distress, and respondents with a score <13 were coded as not having serious psychological distress.

Current cigarette smoking prevalence among adults with inactive epilepsy was higher than that among adults with no history of epilepsy in many of the same subgroups. Current cigarette smoking was also higher among adults with inactive epilepsy than it was among those without epilepsy for any age group; those with <12 or >12 years of education; those with family incomes <100% or >300% of the federal poverty level; among both the insured or the uninsured; the employed; the retired; those in other employment categories (e.g., students); and those residing in all U.S. regions. Among adults without serious psychological distress, cigarette smoking prevalence among those with inactive epilepsy was higher than that among those without epilepsy.

Current cigarette smoking declined significantly among adults without a history of epilepsy, from 19.3% in 2010 to 14.0% in 2017; a 9.3% decline (95% confidence interval [CI] = −10.6% to −7.9%) (p<0.05) and among adults with inactive epilepsy (from 29.2% to 16.2%; a 16.6% decline [95% CI = −31.9% to −1.7%]) (p = 0.03) (Figure). However, declines in current cigarette smoking among adults with active epilepsy were not statistically significant (from 26.4% to 21.8%; a 9.9% decline [95% CI = −23.7% to 3.9%]) (p = 0.2).

**FIGURE:**
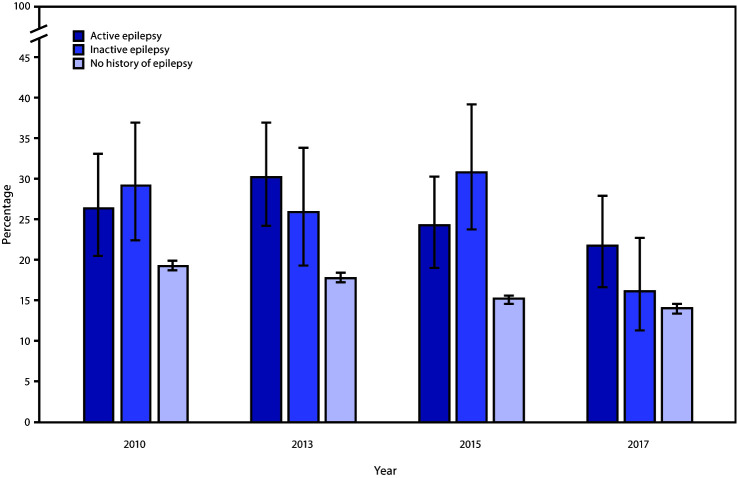
Age-standardized percentage* of current smoking among adults with active epilepsy, inactive epilepsy, and no history of epilepsy, by survey year — United States, 2010, 2013, 2015, and 2017 * With 95% confidence intervals indicated by error bars.

## Discussion

During the 4 survey years (2010, 2013, 2015 and 2017), approximately one in four U.S. adults with active or inactive epilepsy currently smoked cigarettes. This finding reinforces the importance of efforts to reduce cigarette smoking among all adults, especially those with any epilepsy.

Differences in current smoking among adults with epilepsy within subgroups generally paralleled those in the general U.S. adult population, with higher prevalences among some racial/ethnic minorities and those with lower income, a disability, or serious psychological distress (*2*). Like the general population, adults with epilepsy have reported challenges in maintaining healthful behaviors but might benefit from interventions that increase skills for adopting and maintaining healthy behaviors (*8*). Cigarette smoking is especially complex in epilepsy because nicotine and tobacco smoke have both proconvulsant effects (e.g., reducing the anticonvulsive effects of certain antiseizure drugs) and anticonvulsant effects, which has been demonstrated in various human studies and animal models (*5*,*9*). Although one study found that smokers with epilepsy were approximately four times more likely to have experienced a seizure in the past year than were nonsmokers with epilepsy, further research is needed to identify associations between seizure control, current smoking, and smoking cessation in representative samples of persons with active epilepsy (*5*,*9*).

Possible differences in smoking trends between adults with active and inactive epilepsy might be associated with differences in overall health status, work limitations, and quality of life between these two groups (*7*), but this will require further study. Encouraging smoking prevention and cessation among all adults, including those with epilepsy and other population groups with disproportionately higher prevalences of smoking, is critical to reducing their risk of smoking-related disease and death.

The findings in this report are subject to at least six limitations. First, because epilepsy and smoking status were self-reported and not validated by clinical chart review or biochemical testing, these classifications are subject to social desirability bias, interviewer effects, and misclassification of epilepsy. Second, because NHIS excludes institutionalized populations such as the military, detained or incarcerated persons, and nursing home residents, results are not generalizable to these groups. Third, assessment of subgroup differences within a relatively small sample of adults with epilepsy can obscure differences within these populations. Fourth, the NHIS survey response rate has declined from 72% in 2010 to 53% in 2017, resulting in increasing nonresponse bias, which might result in less representative samples of U.S. adults with epilepsy participating in NHIS over time. Fifth, assessment of trends in smoking prevalence among those with active epilepsy might have been underpowered because of sample size limitations. Finally, this study assessed cigarettes only, and not other forms of tobacco products; given that nicotine has been found to have both proconvulsant and anticonvulsant effects, further research on any relationship between epilepsy and the use of noncigarette tobacco products, including e-cigarettes, is warranted.

Health and social service providers who interact with persons with active epilepsy should ensure that smoking cessation information and resources are available to them and should encourage persons who smoke to use these resources to help them quit smoking and to reduce their risk of smoking-related disease and death. Funding state tobacco control programs, including state quit lines, at CDC-recommended levels, increasing tobacco prices, implementing comprehensive smoke-free policies, conducting antitobacco mass media campaigns, and enhancing access to quitting assistance could increase tobacco cessation and reduce tobacco-related disease and death among all adults, including those with epilepsy^††^ (*1*,*10*). Insurers and employers could improve coverage and increase use of cessation treatment, and health systems can integrate cessation interventions into clinical care (*1*).

**Summary**

**What is already known on this topic?**

Studies have shown that cigarette smoking is as common, and sometimes more so, among adults with a history of epilepsy as it is among those without a history of epilepsy.

**What is added by this report?**

During 2010–2017, one in four adults with active or inactive epilepsy were current smokers, compared with one in six persons without epilepsy. Although fewer adults with active epilepsy smoked cigarettes in 2017 than in 2010, this difference was not statistically significant.

**What are the implications for public health practice?**

Epilepsy health and social service providers should promote smoking cessation resources to adults with active epilepsy who smoke cigarettes to help them quit smoking and reduce their risk for smoking-related disease and death.

## Footnotes

* https://www.cdc.gov/tobacco/quit_smoking/cessation/index.htm.

† ftp://ftp.cdc.gov/pub/Health_Statistics/NCHS/Dataset_Documentation/NHIS/2017/srvydesc.pdf.

§ The questions were asked as follows: “Have you ever been told by a doctor or other health professional that you have a seizure disorder or epilepsy?” Responses included Yes, No, Refused, Not ascertained, and Don’t know. If respondents answered “Yes” to having been told that they have seizure disorder or epilepsy, they were also asked “Are you currently taking any medicine to control your seizure disorder or epilepsy?” Responses included Yes, No, Refused, Not ascertained, and Don’t know. Respondents who answered “Yes” were also asked “Think back to last year about the same time. About how many seizures of any type have you had in the past year?” Responses included: None, One, Two or three, Between four and 10, More than 10, Refused, Not ascertained, and Don’t know.

¶ Cigarette smoking percentages were age-standardized to the 2000 U.S. projected population.

** Categorized based on the ratio of total family income to the federal poverty level calculated using the NHIS imputed income files.

†† https://www.cdc.gov/tobacco/data_statistics/sgr/2020-smoking-cessation/index.html.
